# Treatment of hemicrania continua by non-invasive vagus nerve stimulation in 2 patients previously treated with occipital nerve stimulation

**DOI:** 10.1186/1129-2377-1-S14-P230

**Published:** 2013-02-21

**Authors:** A Nesbitt, J Marin, P Goadsby

**Affiliations:** 1 Royal Free London NHS Foundation Trust, UK

## Introduction

Hemicrania Continua is an indomethacin sensitive chronic primary headache syndrome consisting of constant unilateral pain with severe cranial autonomic exacerbations. Long-term indomethacin use can be associated with side effects, resulting in discontinuation of the drug in some patients. Current neuromodulation approaches to treatment, including occipital nerve stimulation (ONS), all require surgical implantation. A novel alternative treatment for this disorder is therefore needed.

We assessed the usefulness of a new non-invasive, portable vagus nerve stimulation (VNS) device, the GammaCore, in two patients who were unable to take indomethacin. Both had previously responded to ONS with the Bion implant[1], but had subsequently had it explanted.

## Results

Patient 1 (male, 61) had been using the device both prophylactically and acutely for 24 weeks at last follow up, with an overall improvement in his baseline condition, estimating a 30% improvement in background pain and 20% improvement in painful autonomic exacerbations. Acutely the device was able to abort exacerbations within 15 minutes most of the time, and it significantly improved the pain for the remainder of attacks. He felt satisfied with the device, although did not feel it was as helpful as the Bion implant.

Patient 2 (female, 56) had been using the device following an exclusively prophylactic regimen for 32 weeks at last follow up, with an overall improvement in her baseline condition, estimating a 75% improvement in both background pain and painful autonomic exacerbations. She felt very satisfied with the device and felt it to be superior to the Bion implant.

Both would recommend non-invasive VNS to other patients.

## Conclusions

This is the first evaluation of an entirely new treatment modality for hemicrania continua, which may offer a non-invasive neurostimulation alternative in selected patients.

Further work is needed to establish this efficacy in a larger series of patients.

## Conflict of interest

This work was supported by an unrestricted grant from ElectroCore Medical, LLC.
